# Impact of donor lung colonized bacteria detected by next-generation sequencing on early post-transplant outcomes in lung transplant recipients

**DOI:** 10.1186/s12879-020-05393-w

**Published:** 2020-09-21

**Authors:** Dong Liu, Ji Zhang, Bo Wu, Feng Liu, Shugao Ye, Hongmei Wang, Jian Lv, Xing Weng, Yan Chen, Weili Han, Jingyu Chen

**Affiliations:** 1 Lung Transplant Center, Wuxi People’s Hospital Affiliated to Nanjing Medical University, Wuxi, 214023 China; 2 BGI Wuhan Biotechnology, BGI-Shenzhen, Wuhan, 430074 China; 3 BGI PathoGenesis Pharmaceutical Technology, BGI-Shenzhen, Shenzhen, 518083 China; 4 Department of Lung Transplantation, First Affiliated Hospital, School of Medical, Zhejiang University, Hangzhou, 310000 China

**Keywords:** Next-generation sequencing, Donor lung, Lung transplantation, Colonized bacteria

## Abstract

**Background:**

The effect of donor lung colonized bacteria on the prognosis of lung transplantation is not clear. We used the technique of next-generation sequencing (NGS) to detect the colonized bacteria from the lower respiratory tract and analyzed whether the colonized bacteria of donor lung could affect the outcomes of lung transplantation.

**Methods:**

Seventeen patients who underwent lung transplantation from March 2018 to June 2018 at Wuxi People’s Hospital affiliated to Nanjing Medical University were included in this study. Twelve cases of donor lung were obtained, and 17 lung transplants were performed, including 12 single lung transplantation and 5 bilateral lung transplantation. The colonized bacteria in the lower lobe tissue of donor lung were detected by NGS, and the bacteria culture method was used to detect the bacteria in the airway secretion before and after the operation. The information of length of extracorporeal membrane oxygenation (ECMO) support, mechanical ventilation time, length of intensive care unit (ICU) stay, duration of fever and length of hospital stay were collected for prognostic analysis.

**Results:**

Compared with bacterial culture methods, the positive rate by using NGS in the lungs were higher (52.9% vs 41.2%). Among the patients who were transplanted with donor lungs with detected bacteria by NGS before surgery, only one patient (1/9) developed the same bacteria after lung transplantation. Based on results of NGS and bacterial culture, there was no association between the colonized bacteria in donor lungs and the patients’ outcomes of immediate posttransplant period.

**Conclusion:**

NGS showed more sensitive than bacterial culture for detection of bacteria. The colonized bacteria in different parts of the lung are inconsistent. There is no association between the colonized bacteria in donor lungs and short-term outcome of lung transplantation patients.

## Background

Currently, in China, lung grafts are often from the donors who were with endotracheal intubation or tracheostomy and mechanical ventilation in the intensive care unit for a long time, increasing the potential risk of infection transmission or bacteria colonization. Therefore, the selection of donor lungs is very important. Even though we had used the lung selection criteria of the Chinese Lung Transplantation Data Center to exclude infection of donor lungs, the colonized bacteria in donor lungs are still existent. Some studies have shown that traditional bacterial culture methods were not sensitive enough. Using routine bacterial culture methods in patients with community-acquired pneumonia, in approximately 20% of specimens from children and 60% of specimens from adults were unable to detect pathogens [1, 2]. Therefore, a new detection method is needed to clarify the colonized bacteria in lungs.

High-throughput pathogenic microbial gene detection technology (also named as Next Generation Sequencing, NGS) is a new technology developed in recent years, which can improve the detection rate of pathogens [3–7]. A randomized, blinded, prospective study suggests that the NGS is highly consistent with traditional methods and is more reliable in terms of negative diagnosis. In a report of 101 patients, NGS and traditional bacterial culture methods were used to detect colonized bacteria in blood. Seventy-two patients (63 negative and 9 positive) had consistent results from the two methods, but the positive rate of NGS in the general population is higher than the traditional methods [8].

At present, there is no conclusion about whether colonized bacteria in donor lungs have effect on the prognosis of lung transplant patients. Therefore, we used NGS to detect the incidents of colonized bacteria in donor lungs, and clarify whether the early prognosis of lung transplant recipients is associated with the colonization of donor lungs.

## Material and methods

### Characteristics of donors and recipients

Donor lungs inclusion criteria: a. Age < 60 years old, smoking history < 20 packs/year. b. No chest injury. c. Continuous mechanical ventilation < 1 week. d. PaO2>300 mmHg (FiO2 = 100%, PEEP = 5 cmH_2_O). e. X-ray or CT shows that the lung field is relatively clear. f. There is no abscess secretion in the lung bronchus at all levels though bronchoscopy.

Donor lungs exclusion criteria: a. Age > 60 years old, smoking history > 20 packs/year. b. Chest trauma and lung contusion. c. Continuous mechanical ventilation > 1 week. d. PaO_2_ < 300 mmHg (FiO_2_ = 100%, PEEP = 5 cmH_2_O). e. X-ray or CT shows that the lung field is infected. f. There are purulent secretions at bronchoscopy in the donor lower airways. g. The percentage of white blood cells, neutrophils, C-reactive protein, and procalcitonin increases gradually comparing with the situation at onset of disease. h. The donor’s body temperature is higher than normal. i. Blood culture is positive.

From March 1st, 2018 to June 30th 2018, we completed 48 lung transplants totally, 17 cases of which were included in the study. The inclusion criteria of the patients included: patients had no pulmonary infection, no mechanical ventilation or tracheotomy; patients met the indications for lung transplantation and there were no contraindications for lung transplantation.

### Sample collection and preservation

According to the National Lung Transplantation Data Center for donor lung selection criteria, this study included 12 donor lungs. 17 lung transplantations were performed, including 12 single lung transplantation and 5 bilateral lung transplantation from March 2018 to June 2018 at Wuxi People’s Hospital affiliated to Nanjing Medical University (Wuxi, China). This study included 12 donor lung tissue samples which were biopsied with a cutting suturing device from the basal segment of the lower lobe, the size of tissues was about 0.5 cm × 0.5 cm. The lung tissue samples were frozen in solid carbon dioxide immediately. NGS is used for bacterial detection of the donor lungs (BGI PathoGenesis Pharmaceutical Technology). Swabs were used to collect the secretions from the bronchi of the donor lungs for bacterial culture. After lung transplantation, sputum was collected from the recipients for bacterial culture. The specimens of lung tissue and bronchial secretions of the donor lung were collected 1 h before transplantation. In order to avoid the influence of secondary infections, the postoperative sputum was collected within 1 week, and the sputum was collected multiple times within 1 week after the operation. This study was approved by Ethics Committee of Wuxi People’s Hospital affiliated to Nanjing Medical University, and each patient has signed an informed consent form.

### Sample detection

The nucleic acid sequence of the pathogenic microorganism in the sample was analyzed, and the microorganism was identified by comparing with the nucleic acid sequence of the existing microorganism in the database. The detection process included: nucleic acid extraction, library construction, sequencing, information analysis and report interpretation.

### Tissue sample: sample processing and DNA extraction

Tissue sample from the donor lung was collected and cut into small pieces according to standard procedures. A 1.5 mL microcentrifuge tube with 0.7 mL lysis buffer, together with pieces of tissue sample and 1 g 0.5 mm glass bead were attached to a horizontal platform on a vortex mixer and agitated vigorously at 2800-3200RPM for 30 min. 0.3 mL sample was separated into a new 1.5 mL microcentrifuge tube and DNA was extracted using the TIANamp Micro DNA Kit (DP316, TIANGEN BIOTECH) according to the manufacturer’s recommendation.

### Construction of DNA libraries

DNA libraries were constructed through DNA-fragmentation, end-repair, adapter-ligation and PCR amplification. Agilent 2100 was used for quality control of the DNA libraries. Quality qualified libraries were sequenced by BGISEQ-50 platform [9].

### Sequencing and bioinformatic analysis

High-quality sequencing data were generated by removing low-quality, and short (length < 35 bp) reads, followed by computational substraction of human host sequences mapped to the human reference genome (hg19) using Burrows-Wheeler Alignment [10]. The remaining data by removal of low-complexity reads were classified by simultaneously aligning to four Microbial Genome Databases, consisting of viruses, bacteria, fungi, and parasites.

The classification reference databases were downloaded from NCBI (ftp://ftp.ncbi.nlm.nih.gov/genomes/). RefSeq contains 4061 whole genome sequence of viral taxa, 2473 bacteral genomes or scaffolds, 199 fungi related to human infection, and 135 parasites associated with human diseases.

### Statistical analysis

Descriptive statistics were computed for the overall sample and stratified by presence of positive bacteria detected by NGS or bacterial culture positive on donor lung samples. Mean ± standard deviation (SD) or median (interquartile range, IQR) was used for describing the continuous variables. We used *t-* test/ANOVA or non-parametric Wilcoxon-Mann-Whitney (for continuous variables) and chi-squared or Fisher’s Exact test for categorical variables to compare the difference between two groups. The significance level was set at 0.05. SPSS 19.0 for Windows (SPSS Inc., Chicago, IL, USA) was used for statistical analysis.

## Results

### Clinical and microbiological characteristics

Details the basic characteristics of the study patients were showed in Table 1. Of the 17 patients in the overall cohort, there were 10 males and 7 females who underwent lung transplantation. Among them, colonized bacteria in donor lung were detected from 6 males and 3 females by NGS. The average age of the overall patients was 52.18 ± 9.5 years, the youngest recipient and the oldest recipient was 31 and 66 years old respectively. The average age of patients who received donor lungs with NGS detected colonized bacteria was 51.33 ± 10.84 years old, with an age range of 31–65 years. On the contrary, the average age of patients who received donor lungs without NGS detected colonized bacteria was 55.25 ± 7.92 years old, with an age range of 47–66 years. Of the 17 lung transplantations, 12 were single lung transplantation and 5 were bilateral lung transplantation. 10 of the 12 donor lungs of the single lung transplantation patients were derived from five donors. In the donor lungs of lung transplant recipients, 7 of the single lung transplantation patients had detected colonized bacteria by using NGS (7/12), and 2 of the donor lungs of the bilateral lung transplantation were detected with colonized bacteria (2/5). In this study, the primary disease of lung transplantation patients were interstitial lung disease (76.5%) and chronic obstructive pulmonary disease (COPD) (23.5%) (Table 1).

**Table 1:** Patient demographics

Patient characteristics	Overall cohort *n* = 17	Donor lungs with colonized bacteria^a^ *n* = 9 (52.9%)	Donor lungs without colonized bacteria^a^ *n* = 8 (47.1%)
Gender, n (%)			
Male	10 (58.8)	6 (66.7)	4 (50)
Female	7 (41.2)	3 (33.3)	4 (50)
Age			
Mean (SD)	53.18 (9.5)	51.33 (10.8)	55.25 (7.9)
Median (min, max)	53 (31,66)	53 (31,65)	55 (47, 66)
Type of transplant, n (%)			
Single lung	12 (70.6)	7 (77.8)	5 (62.5)
Bilateral lung	5 (29.4)	2 (22.2)	3 (37.5)
Underlying condition, n (%)			
ILD	13 (76.5)	6 (66.7)	7 (87.5)
COPD	4 (23.5)	3 (33.3)	1 (12.5)

^a^The colonized bacteria was detected by NGS

### Classification of lung colonized bacteria detected by different methods

We also analyzed the types of lung colonized bacteria by NGS, and the types of bacteria cultured from lung airway secretions of donor lungs and post-transplantation lungs. The proportion of bacteria detected by NGS in donor lungs is 52.9%, and that of bacteria detected by bacterial culture in donor lungs is 35.3%. In only 5 (29.4%) cases, the bacteria detected by NGS in donor lungs with the tissues from the lower part of the lungs were identical to the bacteria cultured in the bronchial secretions of the lungs. Therefore, the colonized bacteria of different parts of the lungs is not consistent, and it is more sensitive to use NGS to detect bacteria than classical bacterial culture method (Table 2).

**Table 2:** Bacteria detected by different methods for each lung transplant patient

Case number	Bacteria detected by using NGS from donor lungs	Bacteria cultured from donor lungs	Bacteria cultured after lung transplantation
1	Non	Non	*Acinetobacter baumannii*
2	*Acinetobacter baumannii,* *Gordon*	Non	Non
3	*Campylobacter, Haemophilus parainfluenzae*	Non	*Klebsiella pneumoniae*
4	*Campylobacter, Haemophilus parainfluenzae*	Non	*Klebsiella pneumoniae*
5	*Rothia*	Non	*Acinetobacter baumannii*
6	Non	Non	Non
7	Non	Non	Non
8	*Streptococcus pyogenes,*	*Serratia,* *Klebsiella oxytosus*	Non
9	*Corynebacterium ammoniagenes,* *Pseudomonas sphaeroides,* *Stenotrophomonas maltophilia*	*Serratia,* *Klebsiella oxytosus*	*Acinetobacter baumannii,* *Klebsiella pneumoniae*
10	Non	*Acinetobacter baumannii*	Non
11	Non	*Acinetobacter baumannii*	*Acinetobacter baumannii,* *Klebsiella pneumoniae*
12	*Candida albicans*	*Candida albicans*	Non
13	*Candida albicans*	*Candida albicans*	*Acinetobacter baumannii*
14	Non	Non	Non
15	Non	Non	*Candida albicans,* *Pseudomonas aeruginosa*
16	*Acinetobacter baumannii,* *Acinetobacter junii,* *Staphylococcus aureus,* *Corynebacterium*	*Acinetobacter baumannii*	*Acinetobacter baumannii*
17	Non	Non	Non

Of the 17 patients, 9 (52.9%) patients were detected with bacteria by sputum culture after operation. In 5 (29.4%) cases, the bacterial detected by NGS were compared with the sputum culture results of post-operation, and only 1 (11.1%) of the patients who detected bacteria by using NGS before operation had the same postoperative bacteria after lung transplantation. It indicated that the bacteria are not mainly derived from colonized bacteria in the donor lung before operation, and may be more closely related to secondary infection (Table 2).

### The transmission of colonized bacteria in lungs before and after operation

For the classification and analysis of the bacterial species which detected under different conditions, we found that NGS could detect more bacteria in the donor lungs, and those bacteria are not specific. The most common type of bacteria found in donor lung bacterial cultures is *Acinetobacter baumannii*. But the types of cultured bacteria after surgery were significantly different from that of the donor lungs before operation. Among them, *A. baumannii* is the most important infectious bacteria (50%), followed by *Klebsiella pneumoniae* (25%) and *Candida albicans* (16.7%). Bacteria detected after lung transplantation derived from secondary infections, showed no relationship with the colonization of bacteria in the donor lungs (Table 3).

**Table 3:** Bacterial species detected by different methods

Pathogenic bacteria	Number of bacteria detected by using NGS from donor lungs, n (%)^a^, Total *n* = 14	Number of bacteria cultured from donor lungs, n (%)^a^, Total *n* = 6	Number of bacteria cultured after lung transplantation, n (%)^a^, Total *n* = 12
*Acinetobacter baumannii*	2 (14.3)	3 (50)	6 (50)
*Candida albicans*	1 (7.1)	1 (16.7)	2 (16.7)
*Klebsiella pneumoniae*	0 (0)	0 (0)	3 (25)
*Pseudomonas aeruginosa*	0 (0)	0 (0)	1 (8.3)
*Gordon*	1 (7.1)	0 (0)	0 (0)
*Campylobacter*	1 (7.1)	0 (0)	0 (0)
*Haemophilus parainfluenzae*	1 (7.1)	0 (0)	0 (0)
*Rotella*	1 (7.1)	0 (0)	0 (0)
*Streptococcus pyogenes*	1 (7.1)	0 (0)	0 (0)
*Corynebacterium ammoniagenes*	1 (7.1)	0 (0)	0 (0)
*Pseudomonas sphaeroides*	1 (7.1)	0 (0)	0 (0)
*Stenotrophomonas maltophilia*	1 (7.1)	0 (0)	0 (0)
*Acinetobacter junii*	1 (7.1)	0 (0)	0 (0)
*Staphylococcus aureus*	1 (7.1)	0 (0)	0 (0)
*Corynebacterium*	1 (7.1)	0 (0)	0 (0)
*Serratia*	0 (0)	1 (16.7)	0 (0)
*Klebsiella oxytosus*	0 (0)	1 (16.7)	0 (0)

^a^Percentages may not add up to 100% due to rounding

### Patient outcomes

We also collected the clinical information and analyzed whether the colonized bacteria in donor lungs could affect the outcome of patients. Patients were divided into group with colonized bacteria (the colonized bacteria in donor lung was positive detected by NGS) and group without colonized bacteria (the colonized bacteria in donor lung was negative detected by NGS). There was no difference in extracorporeal membrane oxygenation (ECMO) support time, mechanical ventilation time, intensive care unit (ICU) stay time, duration of fever and the number of days in hospital between the two groups (Table 4). There was also no correlation between the colonized bacteria in the bronchus of donor lungs and the prognosis of the patients (Table 5). This indicates that there is no significant correlation between the presence of colonized bacteria in donor lungs and the short-term prognosis of the patients.

**Table 4:** Patient outcomes. (Patients with/without colonized bacteria)

Patient outcomes	Overall cohort *n* = 17	Patients with colonized bacteria*, *n* = 9 (52.9%)	Patients without colonized bacteria*, *n* = 8 (47.1%)	*P*-value
**ECMO support time (hours)**				
Mean (SD)	23.8 (50.9)	10.8 (13.0)	38.4 (72.6)	0.322
**Hours on mechanical ventilation (hours)**				
Mean (SD)	73.3 (67.6)	67.1 (72.8)	80.3 (65.4)	0.701
**ICU stay time (hours)**				
Mean (SD)	112.6 (59.8)	98.4 (64.2)	128.5 (53.8)	0.311
**Fever time in one month after surgery (days)**				
Mean (SD)	4.94 (3.9)	4.22 (3.9)	5.75 (4.1)	0.444
**Days in hospital (days)**				
Mean (SD)	41.2 (46.9)	47.6 (25.1)	43.4 (68.4)	0.882

*The colonized bacteria was detected by NGS

**Table 5:** Patient outcomes (Patients with/without culture bacteria)

Patient outcomes	Overall cohort *n* = 17	Patients with culture bacteria*, *n* = 7 (41.2%)	Patients without culture bacteria*, *n* = 10 (58.8%)	*P*-value
**ECMO support time (hours)**				
Mean (SD)	23.8 (50.9)	12.1 (13.7)	31.9 (65.6)	0.375
**Hours on mechanical ventilation (hours)**				
Mean (SD)	73.3 (67.6)	81.3 (77.9)	67.7 (63.1)	0.710
**ICU stay time (hours)**				
Mean (SD)	112.6 (59.8)	119.1 (67.5)	108.0 (57.0)	0.728
**Fever time in one month after surgery (days)**				
Mean (SD)	4.94 (3.9)	3.85 (2.8)	5.70 (4.5)	0.322
**Days in hospital (days)**				
Mean (SD)	41.2 (46.9)	28.8 (25.2)	56.9 (57.6)	0.997

*The bacteria was detected by culture from bronchial secretion in donor lungs

## Discussion

Patients in the ICU have a multi-drug resistant bacteria infection risk due to the abuse of antibiotics and the increase of invasive procedures, especially the carbapenem-resistant *K. pneumoniae* and other carbapenem-resistant Enterobacteriaceae [11, 12] . It was reported that the donors could be infected by multi-drug resistant nosocomial bacteria within 2 days, and spread those bacteria to recipients [13]. Most donor organs are donated by brain-dead patients from ICU. It is common that the donor lungs had been infected and colonized by bacteria, increasing the risk of donor-derived infections. In United States, the total number of donor-derived disease transmission events on the Organs Access and Transplant Network is also increasing year by year, which was believed that donor-derived infections accounted for 71% of infection events post-transplantation. And the probability of disease-specific transmission from the donor to the recipient was different. The probability of malignant tumors, viruses, bacteria, fungi, and parasites caused by donor organs is 67, 46, 34, 29, and 17%. Compared with other organ transplantation, 80% of lung transplant recipients manifested an infection when they received an organ from a same donor who had communicable disease [14]. It was reported that 18 (10.5%) donors were fixed or infected by carbapenem-resistant Gram-negative bacteria in 170 donors who met the transplant criteria in 10 hospitals in Italy during January 1st, 2012 to December 31st, 2013, but those bacteria were not found during donation of organs and transplantation [15]. Therefore, the screening for colonized bacteria or infected pathogens in donor lungs is particularly important.

In China, we currently receive donor lungs in accordance with the donor lung selection criteria of National Lung Transplantation Data Center, based on medical history, chest X-ray or chest CT, fiberoptic bronchoscopy, blood cell counter, C-reactive protein, procalcitonin and other tests to comprehensively assess the infection of the lungs, and exclude the lungs when there are evidence of pulmonary infection. However, we can still detect pathogenic microorganisms from the lungs that meet the standards for lung utilization, which was defined as colonized bacteria. Specimens collected for traditional detecting methods include bronchial secretions or alveolar lavage fluid. In this study, we not only used bronchial secretions for bacteria culture but also lung tissue for NGS, we hoped to evaluate the different distribution of colonized bacteria of the donor lungs comprehensively and the prognosis of recipients were further demonstrated.

It is still unclear whether the pathogenic microorganisms in the donor lungs affect the prognosis of lung transplant recipients. Bonde had reported that 57 of the 64 donors (89%) had positive bacteria for bronchial secretion culture. The study had shown that the colonization of pathogenic microorganisms in donor lungs was common. And multi-factor analysis of pneumonia after lung transplantation found that there was no significant correlation between the culture results of bronchial secretions and pneumonia after lung transplantation. Most symptomatic infections of the recipients were not directly related to the presence of donor organisms. It appeared to be no correlation between organisms identified on donor cultures and pathogenic organisms infected with receptors [16]. Ahmad’s study showed that 32 lung transplant recipients, of which 20 (63%) were positive for bronchial secretion culture, 12 (37%) were culture-negative, and the bronchial secretion positive group had a longer mechanical ventilation time than the negative group. But there was no significant difference in the 30-day survival rate and the incidence of grade 3 primary graft dysfunction (PGD) between the two groups [17]. A study by Avlonitis retrospectively analyzed the culture of lung alveolar lavage fluid in 115 lung transplant recipients, including 53 positive (46%) and negative 62 (54%) for pulmonary alveolar lavage fluid. In the culture-positive recipients, the average tracheal intubation time and ICU intensive care time were longer than the negative group, and the 6-month, 1-year, and 2-year survival rates were lower than the negative group [18].

In this study, the lung colonized bacteria were detected by NGS of lung tissue and bronchial secretion culture. The results of the two methods were not identical due to different sample types. More pathogenic microorganisms could be detected by NGS than traditional culture, and the precise method of gene detection could reflect the distribution of bacteria for donor lung more comprehensive. Although the patients were grouped according to NGS test results or bronchial secretion culture results, the results showed that there was no difference in extracorporeal membrane oxygenation (ECMO) support time, mechanical ventilation time, intensive care unit (ICU) stay time, duration of fever and the number of days in hospital between positive group and negative group. It indicates that neither the bronchial colonized bacteria nor the lung tissue colonized bacteria in donor lungs could affect the early prognosis of lung transplant recipients.

Due to the shortage of standard donor lungs, the use of expanded standard donor lungs was increased in recent years, it was defined as expanded standard donor lungs when bronchoscopy revealed purulent secretion or sputum culture found bacteria [19, 20]. However, whether the expansion of the standard for donor lungs has an impact on the prognosis of recipients is still inconclusive [20, 21]. In fact, positive bacteria culture based on sputum could be caused by lung infection or colonized bacteria. This study shows that the donor lungs which had colonized bacteria can also be used for lung transplantation. If there is no clear clinical evidence for lung infection, pathogenic microorganisms in sputum or bronchial secretions could be just defined as colonized bacteria. There is no significant correlation between the presence of colonized bacteria in donor lungs and the short-term prognosis of the patients. It greatly inspires further research on the stratification and definition of donor lung expansion criteria.

In our research, the colonized bacteria in donor lungs did not cause postoperative pulmonary infection in lung transplant recipients due to the prophylactic application of antibiotics. Previous researches have shown that postoperative targeted antibacterial agents could prevent recipient infections caused by multidrug-resistant Gram-negative bacilli in lungs [22, 23]. In this study, the pathogenic microorganisms of the recipients were generally in tune with the bacteria spectrum in the ICU of our center, and there was no correlation with the colonized bacteria of the donor lungs. It could be caused by colonized bacteria of the donor lungs thus causing subclinical infection or lung injury, making the lungs susceptible to infection by different pathogenic microorganisms of the recipients [24, 25].

There are also limitations in this research. The number of cases enrolled is not enough, and the follow-up time is relatively short. Moreover, the long-term effect of lung colonization on prognosis of lung transplant recipients has not been fully illustrated. Further confirmation is needed for large sample size and long-term follow-ups.

## Conclusion

The incidence of donor-derived infections is still high. Accurate screening of donor-derived pathogen or colonized bacteria is very important to reduce the risk of infection of the recipient. NGS is a new technique for detecting pathogens. We found that it is more sensitive to detect pathogens than traditional bacterial cultures. In our study, the presence of donor colonized bacteria did not affect the early prognosis of recipients. This finding provides the possibility to expand the criteria of donor lungs from bacteria-colonized donors. In the future, large-scale clinical studies are needed to further confirm this conclusion with its impact on the long-term prognosis of recipients.

## Data Availability

The datasets used during the current study are available from the corresponding author on reasonable request.

## Abbreviations

NGS: Next generation sequencing
ICU: Intensive care unit
PGD: Preimplantation genetic diagnosis
ECMO: Extracorporeal membrane oxygenation

## References

[1] Jain S, Williams DJ, Arnold SR (2015). Community-acquired pneumonia requiring hospitalization among U.S. children. N Engl J Med.

[2] Jain S, Self WH, Wunderink RG (2015). Community-acquired pneumonia requiring hospitalization among U.S. adults. N Engl J Med.

[3] Grumaz S, Stevens P, Grumaz C (2016). Next-generation sequencing diagnostics of bacteremia in septic patients. Genome Med.

[4] Decker SO, Sigl A, Grumaz C, et al. Immune-response patterns and next generation sequencing diagnostics for the detection of mycoses in patients with septic shock-results of a combined clinical and experimental investigation. Int J Mol Sci. 2017;18(8):1796. 10.3390/ijms18081796 PMC5578184 28820494

[5] Brenner T, Decker SO, Grumaz S (2018). Next-generation sequencing diagnostics of bacteremia in sepsis (next GeneSiS-trial): study protocol of a prospective, observational, noninterventional, multicenter, clinical trial. Medicine (Baltimore).

[6] Charlson ES, Diamond JM, Bittinger K (2012). Lung-enriched organisms and aberrant bacterial and fungal respiratory microbiota after lung transplant. Am J Respir Crit Care Med.

[7] Gorzer I, Guelly C, Trajanoski S (2010). Deep sequencing reveals highly complex dynamics of human cytomegalovirus genotypes in transplant patients over time. J Virol.

[8] Parize P, Muth E, Richaud C (2017). Untargeted next-generation sequencing-based first-line diagnosis of infection in immunocompromised adults: a multicentre, blinded, prospective study. Clin Microbiol Infect.

[9] Jeon YJ, Zhou Y, Li Y (2014). The feasibility study of non-invasive fetal trisomy 18 and 21 detection with semiconductor sequencing platform. PLoS One.

[10] Li H, Durbin R (2009). Fast and accurate short read alignment with burrows-wheeler transform. Bioinformatics.

[11] Sievert DM, Ricks P, Edwards JR (2013). Antimicrobial-resistant pathogens associated with healthcare-associated infections: summary of data reported to the National Healthcare Safety Network at the Centers for Disease Control and Prevention, 2009-2010. Infect Control Hosp Epidemiol.

[12] Nordmann P, Naas T, Poirel L (2011). Global spread of Carbapenemase-producing Enterobacteriaceae. Emerg Infect Dis.

[13] Martins N, Martins IS, de Freitas WV (2012). Severe infection in a lung transplant recipient caused by donor-transmitted carbapenem-resistant Acinetobacter baumannii. Transpl Infect Dis.

[14] Green M, Covington S, Taranto S (2015). Donor-derived transmission events in 2013: a report of the organ procurement transplant network ad hoc disease transmission advisory committee. Transplantation.

[15] Mularoni A, Bertani A, Vizzini G (2015). Outcome of transplantation using organs from donors infected or colonized with Carbapenem-resistant gram-negative Bacteria. Am J Transplant.

[16] Bonde PN, Patel ND, Borja MC (2006). Impact of donor lung organisms on post-lung transplant pneumonia. J Heart Lung Transplant.

[17] Ahmad O, Shafii AE, Mannino DM (2018). Impact of donor lung pathogenic bacteria on patient outcomes in the immediate post-transplant period. Transpl Infect Dis.

[18] Avlonitis VS, Krause A, Luzzi L (2003). Bacterial colonization of the donor lower airways is a predictor of poor outcome in lung transplantation. Eur J Cardiothorac Surg.

[19] Lardinois D, Banysch M, Korom S (2005). Extended donor lungs: eleven years experience in a consecutive series. Eur J Cardiothorac Surg.

[20] Moreno P, Alvarez A, Santos F (2014). Extended recipients but not extended donors are associated with poor outcomes following lung transplantation. Eur J Cardiothorac Surg.

[21] Smits JM, van der Bij W, Van Raemdonck D (2011). Defining an extended criteria donor lung: an empirical approach based on the Eurotransplant experience. Transpl Int.

[22] Ariza-Heredia EJ, Patel R, Blumberg EA (2012). Outcomes of transplantation using organs from a donor infected with Klebsiella pneumoniae carbapenemase (KPC)-producing K. pneumoniae. Transpl Infect Dis.

[23] Goldberg E, Bishara J, Lev S (2012). Organ transplantation from a donor colonized with a multidrug-resistant organism: a case report. Transpl Infect Dis.

[24] Zenati M, Dowling RD, Dummer JS (1990). Influence of the donor lung on development of early infections in lung transplant recipients. J Heart Transplant.

[25] Dowling RD, Zenati M, Yousem SA (1992). Donor-transmitted pneumonia in experimental lung allografts. Successful prevention with donor antibiotic therapy. J Thorac Cardiovasc Surg.

